# Biosafety implications of cadaver preservation methods in veterinary anatomy education

**DOI:** 10.1007/s11259-026-11378-y

**Published:** 2026-06-26

**Authors:** Rui Alvites, Sónia Sá, Mariana Caipira Lei, Sandra Quinteira, Carla Miranda

**Affiliations:** 1 University Institute of Health Sciences (IUCS), Polytechnic and University Higher Education Cooperative (CESPU), Avenida Central de Gandra, Gandra, 1317, 4585-116 Portugal; 2 Applied Molecular Biosciences Unit, UCIBIO, University Institute of Health Sciences (1H-TOXRUN, IUCS-CESPU), Rua Central de Gandra, Gandra, 1317, 4585- 116 Portugal; 3 Associate Laboratory i4HB - Institute for Health and Bioeconomy, University Institute of Health Sciences CESPU, Rua Central de Gandra, Gandra, 1317, 4585-116 Portugal; 4 Associated Laboratory for Green Chemistry (REQUIMTE-LAQV), Institute of Sciences, Technologies and Agro-environment of the University of Porto (ICETA), Rua D. Manuel II, Apartado 55142, Porto, 4051-401 Portugal; 5 Department of Veterinary Clinics, Abel Salazar Institute of Biomedical Sciences (ICBAS), University of Porto (UP), Rua de Jorge Viterbo Ferreira, nº 228, Porto, 4050-313 Portugal; 6 Centre for the Research and Technology of Agro-Environmental and Biological Sciences (CITAB), University of Trás-os-Montes e Alto Douro (UTAD), Quinta de Prados, Vila Real, 5000-801 Portugal; 7 CIBIO/InBIO—Research Center in Biodiversity and Genetic Resources/Research Network in Biodiversity and Biodiversity and Evolutionary Biology, Associated Laboratory, University of Porto, Campus de Vairão, Rua Padre Armando Quintas 7, Vairão, 4485-661 Portugal; 8 BIOPOLIS Program in Genomics, Biodiversity and Land Planning, Campus de Vairão, Rua Padre Armando Quintas 7, Vairão, 4485-661 Portugal; 9 Department of Biology, Faculty of Sciences, University of Porto, Rua do Campo Alegre s/n, Porto, 4169-007 Portugal; 10 Associated Laboratory for Green Chemistry (REQUIMTE-LAQV), University NOVA of Lisbon, Caparica, 1099-085 Portugal

**Keywords:** Veterinary Anatomy, Cadaver Preservation, Saturated Saline Solution, Biosafety, Microbiological Contamination

## Abstract

Cadaver dissection remains fundamental in veterinary medicine and anatomy education, providing an irreplaceable experiential learning opportunity. It fosters three-dimensional anatomical understanding, the development of manual dissection skills, and early insights into clinical practice. Despite growing availability of digital and virtual simulation tools, preserved animal cadavers remain essential and are still preferred for anatomy teaching, surgical training, and diagnostic instruction. No alternative method reproduces anatomical structures with the same fidelity as a real cadaver. Preservation methods significantly affect teaching quality, occupational health for students, instructors, and staff, laboratory biosafety, and regulatory compliance. Traditional formaldehyde fixation effectively prevents tissue degradation and microbial growth; however, it is toxic, potentially carcinogenic, and impairs tissue color, flexibility, and realism. Saturated saline solutions have emerged as a low-cost, accessible, and pedagogically advantageous preservation option. The hyperosmotic environment delays autolysis and putrefaction while better preserving tissue texture, color, and joint mobility. However, saline does not sterilize cadavers, raising concerns about the potential persistence of halotolerant microorganisms, environmental contamination, and occupational exposure risks. This review synthesizes the literature on cadaver preservation in veterinary anatomy, focusing on biosafety and microbiological hazards. Emphasis is placed on preservation in saturated saline solution as a potentially promising alternative method, although long-term microbiological evidence remains limited and fragmented. The review examines evidence of bacterial and fungal contamination in cadavers, preservative solutions, laboratory surfaces, and fomites, particularly clinically relevant and potentially zoonotic pathogens. By integrating perspectives from anatomy education, microbiology, and occupational health, the review identifies knowledge gaps and highlights the need for standardized microbiological monitoring and biosafety protocols in laboratories adopting innovative preservation strategies.

## Introduction

Anatomy is a fundamental discipline in veterinary medicine education, enabling an understanding of structure-function relationships that will later be essential for diagnosis, surgical approaches, and clinical decision-making (Abdullah et al. 2021). Mastery of anatomical knowledge allows students to interpret imaging studies, perform invasive procedures safely, and understand species-specific variations that influence therapeutic approaches. Despite curricular reforms and pedagogical innovations, cadaver dissection remains central to veterinary anatomy education (Clifton et al. 2020).

Over the past two decades, veterinary education has increasingly integrated digital resources, including 3D models, virtual dissection platforms, and augmented or mixed reality tools (Choudhary and Sarkar 2025). These technologies offer obvious visualization advantages and allow for repetitive practice without cadaver degradation in a comfortable and hygienic manner. However, they cannot fully replicate the tactile experience, the complete comprehension of tissue characteristics and planes, and the variability observed during properly monitored and supervised real dissection (Varner et al. 2021).

Veterinary anatomy is grounded in a comparative study of different species, requiring students to understand the musculoskeletal, visceral and neuroanatomical differences among domestic animals of veterinary interest, including companion, wild and production animals. Cadavers remain the most ethically acceptable and pedagogically effective method for exposing students to this diversity, respecting the principles of the 3Rs, such as replacement, reduction and refinement (Terrado et al. 2023).

The effective and long-lasting preservation of cadavers, while maintaining their physical characteristics, resistance, and coloration for extended periods, represents a significant logistical and scientific challenge. The methods selected for preservation must prevent or delay autolysis, maintain the realistic appearance of tissues, minimize health risks to students, teachers, and staff, and ensure environmental safety and economic viability (Tamayo-Arango and Garzón-Alzate 2018). Historically, fixation with formaldehyde was preferred due to its effectiveness as a long-term preservative (Demiryurek et al. 2002; Kaliappan et al. 2023). However, increasing evidence of its adverse effects has driven the search for formaldehyde-free preservation techniques, including alcohol-based, glycerin-based, Thiel-based, and saturated saline solutions (de Oliveira 2014; Lombardero et al. 2017).

Saturated saline solutions (approximately 26–30% NaCl) are gaining prominence in veterinary teaching. They are low-cost, readily available, and effectively preserve tissue texture, color, and joint mobility. However, unlike formaldehyde, they do not sterilize cadavers, allowing halotolerant microorganisms to persist and potentially compromising biosafety (Torres et al. 2018).

Despite the increasing adoption of formaldehyde-free preservation techniques, particularly saturated saline solutions, the microbiological risks associated with these methods remain insufficiently characterized. Existing studies are fragmented, and systematic biosafety frameworks for veterinary and human anatomy laboratories are lacking. Although saturated saline solutions are increasingly regarded as a promising low-cost alternative with notable pedagogical advantages, the long-term microbiological evidence, particularly in veterinary cadavers, remains limited. This review critically evaluates preservation techniques, microbial persistence, and biosafety implications, with particular focus on saturated saline-preserved cadavers. The conceptual contribution of this review lies in its integrated analysis of three distinct but interconnected domains: educational realism, occupational chemical safety, and microbiological biosafety. Those are frequently discussed in isolation. By critically examining saturated saline preservation through this multi-dimensional lens, we aim to provide a practical framework to guide veterinary anatomy laboratories in balancing pedagogical quality with safety when adopting formaldehyde-free methods.

### Search strategy and selection criteria

This is a narrative review aimed at synthesizing the existing literature on cadaver preservation techniques in veterinary anatomy education, with particular emphasis on biosafety and microbiological implications of saturated saline solutions. Literature searches were conducted in PubMed, Scopus, Web of Science, and Google Scholar from November 2025 to March 2026. Search terms included combinations of (“cadaver preservation” OR “embalming” OR “saturated saline solution” OR “sodium chloride” OR “formaldehyde-free”) AND (“veterinary anatomy” OR “biosafety” OR “microbiological contamination” OR “occupational health” OR “zoonotic”). No date restrictions were applied. Peer-reviewed articles, previous reviews, and technical reports in English were included.

Exclusion criteria comprised conference abstracts, non-peer-reviewed sources, retracted articles and studies focused solely on virtual teaching or plastination without any preservation component. Reference lists of selected articles were manually screened for additional relevant publications. Due to the heterogeneity of the literature and the relatively recent adoption of saline-based methods in veterinary education, a formal systematic review following PRISMA guidelines was not performed. Instead, a qualitative synthesis was conducted, with emphasis on study design, direct relevance to veterinary cadavers, and methodological limitations. As a narrative review, this work does not claim to be exhaustive or free from selection bias and lacks the reproducibility of a systematic review. However, this methodological approach was chosen to allow a broader, more critical, and integrated discussion of the topic, encompassing pedagogical, chemical safety, and microbiological perspectives that would be more restricted in a purely systematic approach. Evidence was informally graded according to its applicability to veterinary anatomy (direct veterinary saline studies > human cadaver studies > environmental/extrapolated data). Whenever possible, priority was given to studies performed on veterinary cadavers. Findings from human anatomy or general environmental microbiology are explicitly identified as indirect evidence.

## Cadaver preservation in veterinary anatomy education

### Historical perspective

The use of cadavers in anatomy education has a long and complex history, closely linked to the evolution of medical and veterinary sciences, advances in preservation techniques, and changes in ethical and regulatory frameworks over time. Early anatomical studies relied on recently deceased bodies, refrigeration, or simple drying methods, which offered limited protection against autolysis and microbial decomposition, and severely restricted the duration and quality of practical teaching (Da Silva et al. 2004; Ghosh 2015).

The systematic dissection of corpses became definitively established in Europe during the late Middle Ages and the Renaissance, following the gradual relaxation of religious and legal prohibitions. Anatomists such as Vesalius emphasized direct observation of human and animal bodies, highlighting the need for preservation methods that could prolong dissection time and improve structural visualization. Early preservation strategies included immersion in alcohol, vinegar, brine, or herbal extracts, which slowed decomposition but did not prevent microbial growth or tissue degradation over extended periods (Persaud et al. 2014).

A significant turning point occurred in the late 19th century with the introduction of chemical fixatives. In 1894, Ferdinand Blum described the use of formaldehyde as a tissue fixative, marking the beginning of its widespread adoption in anatomical and histological practice. Formaldehyde quickly became the standard for cadaver preservation due to its strong antimicrobial properties, ability to inhibit enzymatic autolysis, and capacity to maintain structural integrity for extended periods of storage (Lingnau 1957; Demiryurek et al. 2002). Throughout the 20th century, formaldehyde-based embalming dominated the teaching of human and veterinary anatomy, allowing for the repeated use of cadavers across different classes and supporting the expansion of comparative anatomy curricula. Despite its effectiveness, the limitations and dangers of formaldehyde fixation have gradually become evident. Formaldehyde alters and tends to lighten tissue color, increases its stiffness, reduces joint mobility, and produces strong odors, negatively affecting the realism required for surgical training and procedure simulation (Nam et al. 2020).

From the mid-20th century onwards, several alternative methods to formaldehyde fixation were explored to address its toxicity and limitations in tissue realism. These include alcohol-based techniques (using ethanol or isopropanol), introduced as early alternatives with reduced toxicity (Brenner 2014; Balta et al. 2019); glycerin-based methods, which emerged to enhance flexibility and color retention, often combined with antimicrobial agents (Da Silva et al. 2004; de Oliveira 2014); soft embalming techniques, particularly the Thiel method, developed in the late 1980s (first described in 1992) (Thiel 1992) and simple, low-cost approaches such as saturated saline solutions, whose systematic evaluation as formaldehyde-free options for anatomy teaching gained momentum in the early 21 st century (Lombardero et al. 2017).

These methods have been investigated for their potential in veterinary and human anatomy education, with varying degrees of success in preserving tissue properties suitable for dissection and surgical simulation. However, unlike fixation with formaldehyde, other preservation methods like preservation in saline solutions do not sterilize corpses. Historical and contemporary studies indicate that halotolerant bacteria and environmental microorganisms can persist in environments with high salt concentrations, raising biosafety concerns related to occupational exposure and environmental contamination (de Oliveira 2014; Torres et al. 2018). Consequently, the focus of cadaver preservation has evolved from simply preventing decomposition to a more integrated approach that balances educational realism, chemical safety, microbiological risk, and regulatory compliance. In this context, saturated saline solutions represent the latest stage in a long spectrum of preservation strategies, reflecting modern priorities in veterinary anatomy education: minimizing exposure to toxic substances, maintaining pedagogical effectiveness, and addressing biosecurity through complementary monitoring and hygiene protocols, rather than relying exclusively on chemical sterilization.

### Formaldehyde-based fixation

Formaldehyde-based fixation (embalming) has long been the standard method for preserving cadavers in human and veterinary anatomy, mainly due to its chemical ability to stabilize tissues and simultaneously prevent microbial growth (Lingnau 1957; Thiel 1992). Chemically, formaldehyde reacts with the amino groups of proteins to form methylene bridges (-CH₂-), creating extensive cross-links between protein molecules. This cross-linking stabilizes the tissue architecture, preventing enzymatic autolysis and inhibiting the proliferation of bacteria and fungi, allowing cadavers to be stored for long periods and used repeatedly in teaching sessions (Fox et al. 1985; Varner et al. 2021). In veterinary education, this chemical stability ensures that musculoskeletal, visceral, and neuroanatomical structures maintain their preserved shape and spatial relationships, which is essential for easier dissection and for comparative anatomy studies in multiple species (Nam et al. 2020). The formation of protein-protein and protein-nucleic acid cross-links also reduces water activity in tissues, contributing to microbial inhibition and delaying decomposition.

Despite these advantages, formaldehyde fixation has several limitations. Chemically, formaldehyde is highly reactive and toxic; its inhalation or skin contact can cause mucous membrane irritation, respiratory symptoms, and, with chronic exposure, carcinogenic effects (Fox et al. 1985; Lyon 1994). More importantly, growing evidence has demonstrated significant occupational health risks, including mucosal irritation, respiratory symptoms, and carcinogenic potential after chronic exposure (Lyon 1994; Kaliappan et al. 2023). These concerns have motivated regulatory scrutiny and stimulated the search for alternative preservation strategies that can balance educational value with biosafety. From a pedagogical point of view, protein cross-linking increases tissue stiffness, reduces joint mobility, alters and tends to lighten tissue color, and produces strong odors, negatively affecting the realism required for surgical training and procedure simulation (Thiel 1992; Nam et al. 2020). Its pungent odor can also affect student comfort during prolonged dissections, increasing reluctance towards dissection. Furthermore, formaldehyde-based fixation necessitates advanced ventilation and local exhaust systems to maintain airborne concentrations below occupational exposure limits. This typically requires dedicated downdraft tables, local exhaust ventilation, or integrated building extraction systems, representing a substantial investment in equipment and infrastructural modifications (Waschke et al. 2019; Zdilla 2021). Despite these chemical and physical disadvantages, formaldehyde remains widely used where long-term structural preservation and microbial control are priorities, highlighting the need to balance pedagogical effectiveness with chemical safety in veterinary anatomy laboratories.

### Alternative preservation methods

In response to the toxicological and pedagogical limitations associated with formaldehyde-based fixation, several alternative preservation methods of cadaver preservation have been developed for anatomy education. These approaches aim to reduce exposure to chemicals while preserving tissue properties relevant to dissection and basic procedural training, although none fully replicate the antimicrobial efficacy of formaldehyde (Thiel 1992; Nam et al. 2020).

Alcohol-based methods, typically using ethanol or isopropanol, preserve tissues primarily through protein denaturation and dehydration, limiting enzymatic activity and microbial growth. While less hazardous than formaldehyde, prolonged exposure often results in tissue shrinkage, increased stiffness, and color alteration, which can compromise anatomical realism (Brenner 2014; Balta et al. 2019).

Glycerin-based preservation improves tissue flexibility and color retention due to its hygroscopic properties, making specimens more suitable for repeated handling. However, glycerin does not possess intrinsic antimicrobial activity and therefore requires combination with other preservatives to control microbial proliferation, increasing the complexity of the procedure (Da Silva et al. 2004; de Oliveira 2014).

Gentle embalming techniques, particularly the Thiel soft embalming method (introduced by Walter Thiel in 1992), employ complex chemical formulations to achieve tissue flexibility, joint mobility, and overall realism remarkably close to that of a live cadaver. These methods provide superior preservation of natural color, texture, and pliability, making them highly valued, and often considered ideal, for surgical training and procedures requiring realistic tactile feedback. However, their complexity (involving multiple chemical components and precise protocols), high cost, laborious preparation, and dependence on specialized infrastructure have limited widespread and routine adoption, especially in veterinary anatomy education and institutions with constrained resources (Thiel 1992; Eisma et al. 2013).

Alongside these developments, a renewed interest has arisen in simple and low-cost preservation approaches, particularly saturated saline solutions, which have gained prominence more recently as formaldehyde-free preservation alternatives. Their preservative effect relies on hyperosmotic conditions created by high concentrations of sodium chloride, which reduce water availability for enzymatic reactions and microbial metabolism, thereby retarding putrefaction. Although saline preservation techniques have historical precedents in various forms, their systematic evaluation and application as viable options for teaching anatomy gained momentum in the early 21 st century (de Oliveira 2014; Lombardero et al. 2017). Direct studies using veterinary cadavers have demonstrated favorable preservation of tissue color, texture, and joint mobility, making them particularly attractive for veterinary anatomy education (Lombardero et al. 2017). Additionally, saturated saline solutions produce negligible volatile emissions, thereby reducing or eliminating the need for costly and complex ventilation infrastructure required with formaldehyde (Zdilla 2021; Handady et al. 2024). However, they do not fully sterilize cadavers, and halotolerant microorganisms may persist, necessitating complementary biosecurity and hygiene measures to ensure safe handling (Demiryurek et al. 2002; Torres et al. 2018). It should be noted that most microbiological evidence regarding persistence of halotolerant organisms comes from supporting or indirect sources, including human cadaver studies and general environmental microbiology. Nevertheless, quantitative data from veterinary cadavers (Pereira et al. 2019) indicate relatively low microbial loads, and direct evidence of transmission to handlers or clinical infections remains very limited.

In addition to the wet chemical approaches described above, other alternative preservation techniques warrant mention complementary or specialized options in veterinary anatomy education. Plastination, a process developed by Gunther von Hagens in 1977, involves replacing tissue fluids and lipids with curable polymers (such as silicone, epoxy, or polyester), yielding dry, odorless, non-toxic, durable, and highly realistic specimens that are safe for long-term handling, storage, and transport without biohazard risks (Latorre et al. 2007; Yunus et al. 2022; Senos 2024). This method excels in producing permanent anatomical models (whole cadavers, organs, or slices) suitable for self-study, museum displays, or supplementary teaching, and has been applied successfully to equine, canine, and other veterinary specimens, with positive student feedback on realism and accessibility (Latorre et al. 2016; Senos 2024). However, its main disadvantages include high initial costs, specialized equipment and technical expertise requirements, and reduced tissue flexibility, resulting in rigid specimens that limit active wet dissection and joint mobility. Similarly, freezing (typically at −18 °C or lower) or short-term refrigeration (around 2–4 °C) serves as a temporary preservation strategy, often as an initial step or for maintaining near-natural tissue texture and flexibility during surgical skill training or brief dissection sessions. It avoids chemical toxicity and preserves organoleptic properties (Da Silva et al. 2004; Varner et al. 2021). Nevertheless, it is limited by the need for cold storage infrastructure, short shelf-life after thawing, potential tissue damage or increased friability from repeated freeze-thaw cycles, and inability to support long-term repeated use in laboratory settings. These techniques offer valuable advantages in specific contexts, particularly for biosafety and realism, but involve trade-offs in cost, logistical demands, and suitability for repeated wet dissection compared to low-cost, immersion-based alternatives like saturated saline solutions.

**Fig. 1:**
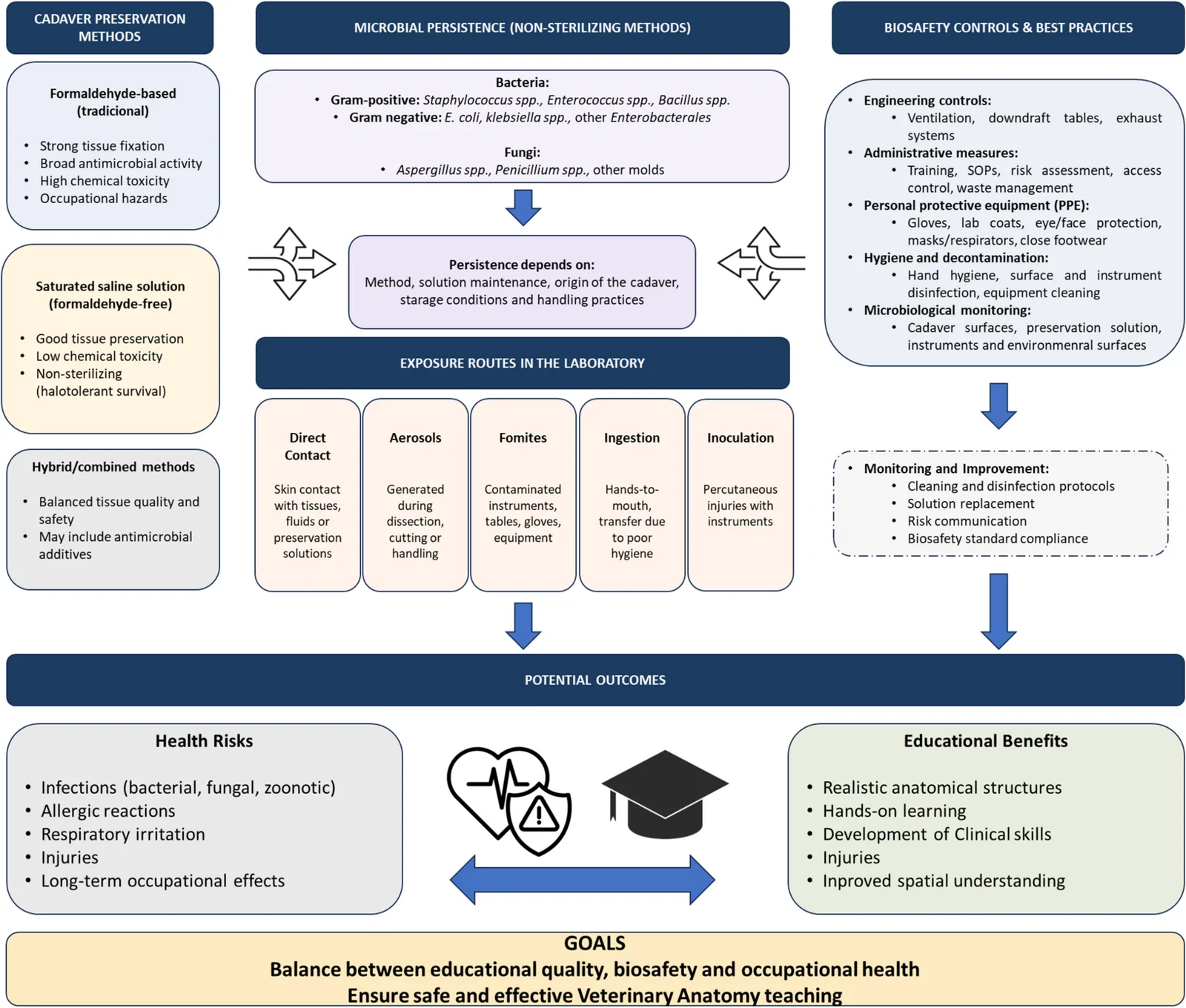
Conceptual framework for cadaver preservation methods in veterinary anatomy education, integrating educational realism, chemical safety, and microbiological biosafety, along with exposure routes and biosafety control measures. The framework highlights trade-offs associated with formaldehyde-free methods such as saturated saline preservation

In general, alternative preservation methods reflect a shift towards a balance between chemical safety, educational effectiveness, and biosecurity management, with method selection depending on institutional resources and teaching objectives. A comparative overview of preservation methods and their associated microbiological risks is presented in Table 1, illustrating the trade-offs between chemical safety and microbial control that underline current preservation strategies. Figure 1 provides a conceptual framework integrating the three main domains (educational realism, occupational chemical safety, and microbiological biosafety), main exposure routes, and recommended biosafety controls when using saturated saline and other formaldehyde-free preservation methods.

**Table 1:** Comparison of cadaver fixation and preservation methods used in veterinary anatomy education, highlighting key advantages, limitations, and microbiological risk

Method	Main advantages	Main limitations	Microbiological risk	Type of Evidence for Microbiological Risk	Available Microbial Load Data
Formaldehyde-based	Long-term preservation; broad antimicrobial activity	Toxic, carcinogenic; reduced tissue flexibility and altered color	Low microbiological risk; however, associated with significant occupational chemical exposure	Mostly Indirect (human and veterinary)	Very low/often below detection limit
Alcohol-based	Lower toxicity than formaldehyde; acceptable structural preservation	Tissue dehydration; need for repeated rehydration	Moderate; risk increases with prolonged storage or inadequate antiseptic supplementation	Indirect (based on general antimicrobial mechanism data; limited veterinary-specific studies)	Limited quantitative CFU/mL data available
Glycerin-based	Soft, flexible tissues; good color retention	Limited antimicrobial activity; requires additives	Moderate to high unless combined with antimicrobial additives	Indirect (limited direct microbiological quantification; mainly inferred from preservation characteristics)	Limited quantitative CFU/mL data available
Thiel embalming	High tissue realism; preserved joint mobility	Complex preparation; high cost	Low when protocols are correctly implemented	Mostly Indirect (primarily human anatomical data with limited veterinary extrapolation)	Limited quantitative CFU/mL data available
Saturated saline solution	Low cost; good preservation of color and texture	Non-sterilizing; limited long-term data	Moderate: potential persistence of halotolerant organisms	Direct (limited veterinary-specific microbiological evidence)	Low counts (≤ 9 × 10¹ CFU/mL) up to 120 days
Plastination	Dry, odorless, non-toxic, durable specimens; excellent for long-term storage, handling without PPE, and 3D realism (ideal for museum/display/supplementary study)	High initial cost and equipment needs; time-consuming; rigid/dry tissues; limits wet dissection and joint mobility	Very low, near-zero after process; no biohazard risk in final specimens	Direct and Indirect (validated sterilization process with post-processing biological inactivity)	No microbial growth (post-process sterile/biologically inactive state)
Freezing/Refrigeration	Preserves near-natural texture, color, and flexibility (excellent for short-term surgical training or as preparatory step); no chemical toxicity	Temporary/short shelf-life post-thaw; requires cold storage infrastructure; potential tissue damage from freeze-thaw cycles	Moderate to high putrefaction risk if not properly managed; microbial growth possible during thawing/storage	Direct (based on observed microbial growth during thawing and storage conditions)	No consistent quantitative CFU/mL data; microbial growth reported during thawing if improperly managed

CFU – colony forming unit. Adapted from (Thiel 1992; Eisma et al. 2013; Brenner 2014; de Oliveira 2014; Lombardero et al. 2017; Balta et al. 2019; Nam et al. 2020; Varner et al. 2021)

The conceptual framework (Fig. 1) differentiates three distinct evidence domains that are frequently addressed together: educational realism, occupational chemical safety, and microbiological biosafety. These domains are interrelated but not interchangeable, and each preservation method presents different strengths and weaknesses across them.

Educational realism concerns the ability of the preserved cadaver to maintain natural color, texture, tissue pliability, and joint mobility, thereby providing students with an optimal hands-on learning experience. Saturated saline solutions perform particularly well in this domain, showing superior preservation of macroscopic tissue characteristics compared to formaldehyde (Lombardero et al. 2017; Nam et al. 2020). Occupational chemical safety evaluates the toxicity and exposure risk of the preservative agents to students, instructors, and staff. In this aspect, saturated saline solutions offer a clear advantage by eliminating volatile toxic compounds and reducing the need for specialized ventilation systems, in contrast to the well-documented hazards of formaldehyde (Zdilla 2021; Handady et al. 2024). Microbiological biosafety, however, constitutes a separate domain focused on the potential survival and transmission of microorganisms. Saturated saline solutions do not exert sterilizing effects and may allow the persistence of halotolerant bacteria and other environmental microorganisms, representing a relative increase in microbiological risk compared to traditional formaldehyde fixation (Torres et al. 2018; Pereira et al. 2019).

These domain-specific trade-offs should be carefully considered when selecting a preservation method. Saturated saline solutions provide strong benefits in educational realism and chemical safety at the cost of greater challenges in microbiological control. Conversely, formaldehyde offers robust microbiological suppression but performs poorly in the other two domains. No single method is optimal in all three areas simultaneously. Therefore, the adoption of non-sterilizing alternatives such as saturated saline requires the implementation of complementary biosafety measures, including routine microbiological monitoring, strict hygiene protocols, and regular environmental surveillance, to ensure an acceptable balance between pedagogical quality and laboratory safety.

## Microbial survival and contamination in preserved cadavers

Microbiological safety is a critical component of cadaver preservation in anatomy teaching, particularly in the context of the increasing adoption of formaldehyde-free preservation methods. Although preservation techniques are designed to delay tissue degradation and reduce microbial activity, preserved cadavers are not sterile and may harbor viable microorganisms originating from the animal’s endogenous microbiota, the surrounding environment, or repeated human handling during teaching activities (CORREA 2003; Molina et al. 2019). Consequently, preserved specimens may represent potential sources of microbial dissemination and occupational exposure if adequate biosafety measures are not implemented.

### Sources of microbial contamination

Following death, microorganisms from the gastrointestinal tract and other endogenous communities spread into tissues and body cavities through a process known as post-mortem microbial succession. This occurs as physiological barriers break down. Although preservation methods aim to halt decomposition processes, residual microbial populations may persist depending on the preservation technique and storage conditions.

Among the microorganisms most frequently reported in anatomy teaching settings, *Enterococcus* spp., *Escherichia coli*, and *Staphylococcus aureus* are particularly relevant (Manero and Blanch 1999; Tabaac et al. 2013; Kosif and Avcioglu 2018). These organisms often serve as indicators of fecal contamination, skin contact, and indirect transmission via fomites, and some strains may exhibit antimicrobial resistance (Manero and Blanch 1999; Kosif and Avcioglu 2018). Their presence has been documented not only on cadaver surfaces but also on dissection tables, surgical instruments, gloves, and other laboratory equipment, highlighting the potential for cross-contamination within the teaching environment.

In veterinary anatomy laboratories, additional considerations arise from the potential presence of zoonotic microorganisms originating from animal cadavers. Although the risk varies depending on the health status of the animal prior to death and the preservation method employed, pathogens such as *Salmonella* spp., *Clostridium* spp., *Listeria monocytogenes*, and *Mycobacterium* spp. have been identified as potential hazards in animal tissues used for educational purposes.

### Survival of microorganisms under different fixation and preservation methods

Preservation methods vary considerably in their ability to control microbial survival. Formaldehyde fixation substantially reduces microbial viability through protein cross-linking and enzyme inactivation, which disrupts essential cellular processes and limits microbial proliferation. Nevertheless, complete sterility cannot always be guaranteed particularly in deeper tissues or when fixation is incomplete (Demiryurek et al. 2002). This evidence derives mainly from human cadaver studies and should be considered indirect for veterinary applications. Alternative preservation methods may exert different levels of antimicrobial activity. Saturated saline solutions, for example, inhibit many microorganisms by reducing water activity and inducing osmotic stress. However, such solutions do not exert a true sterilizing effect. Halotolerant microorganisms, particularly *Enterococcus* spp., can survive in hyperosmotic saline environments, representing constant biosafety concerns during repeated cadaver handling (Torres et al. 2018). Direct evidence from veterinary cadavers preserved in saturated saline remains limited, although available studies (Pereira et al. 2019) support the persistence of halotolerant bacteria. This study reported low bacterial counts (≤ 9 × 10¹ CFU/mL for total aerobes and anaerobes) in canine cadavers preserved in saturated saline solution for up to 120 days, although halotolerant and spore-forming organisms were still detected. However, it should be noted that the existing evidence primarily demonstrates microbial persistence rather than confirmed transmission or infection risk to students and staff. Direct epidemiological data linking saline-preserved cadavers to occupational infections remain very limited.

Fungal contamination, although less frequently reported than bacterial contamination, has also been documented in preserved cadavers. Filamentous fungi such as *Aspergillus* spp. and *Penicillium* spp. may persist on tissue surfaces or in the surrounding laboratory environment, particularly under conditions of high humidity or prolonged storage. These organisms may contribute to allergic reactions, respiratory irritation, or opportunistic infections in susceptible individuals (CORREA 2003; Molina et al. 2019; Kwizera and Naluzze 2024).

### Environmental dissemination in anatomy laboratories

The microbiological risk of cadaver handling extends beyond the specimens to the entire laboratory environment. Microorganisms can spread through direct contact, contaminated instruments, and high-touch surfaces, contaminating dissection tables, tools, and protective clothing. Mechanical manipulation of tissues during dissection may also generate bioaerosols containing viable microorganisms or microbial fragments. These particles can remain suspended in the air and contribute to indirect exposure through inhalation or deposition on environmental surfaces. Consequently, repeated dissection activities may facilitate the circulation of microorganisms within the laboratory ecosystem. Environmental dissemination of microorganisms is a recognized concern in anatomy laboratories warranting appropriate hygiene and control measures (Seçgin et al. 2024).

Empirical studies conducted in anatomy teaching settings consistently demonstrate that preserved cadavers and associated laboratory materials can harbor diverse microbial communities (Tabaac et al. 2013; Molina et al. 2019; Gundreddy and Gaurkar 2022). Similar findings have been reported in anatomy laboratories where microorganisms were isolated from cadaver surfaces, internal tissues, and preservation fluids (Pereira et al. 2019; Seçgin et al. 2024; Streich et al. 2025).

In addition to cadaver tissues, contamination has also been documented in the broader educational environment. Kosif and Avcioglu (Kosif and Avcioglu 2018) investigated bacterial contamination in anatomical teaching materials and demonstrated that indirect exposure pathways may exist even in simulation-based learning environments. Repeated handling of educational materials, shared laboratory equipment, and inadequate disinfection practices may therefore contribute to the persistence and dissemination of microorganisms within anatomy teaching facilities.

Occupational exposure risks associated with handling cadavers were emphasized by Hassan et al. (Ashfaq Ul Hassan 2022), who documented the potential for microbial transmission from cadavers to students, teaching staff, and paramedical personnel. Their findings underscore that the risk is not limited to direct contact with biological tissues but may also involve indirect transmission routes such as contaminated surfaces, fomites, and bioaerosols generated during dissection procedures. In veterinary contexts, this risk may be further amplified by the potential presence of zoonotic pathogens or antimicrobial-resistant microorganisms originating from animal cadavers.

Collectively, these case studies and empirical observations indicate that microbiological safety in anatomy laboratories depends not only on the preservation method itself but also on appropriate laboratory management practices. Formaldehyde-free preservation techniques, including saturated saline solutions, may offer pedagogical and occupational health advantages, yet they must be accompanied by rigorous biosafety measures. Routine surface disinfection, appropriate use of personal protective equipment, controlled handling procedures, and regular microbiological monitoring are essential to mitigate occupational and environmental health risks. Without such measures, the pedagogical benefits associated with alternative preservation techniques may be offset by increased microbiological and biosafety concerns.

## Biosafety in veterinary anatomy laboratories

### Types of laboratory hazards

Anatomy laboratories constitute complex occupational environments in which biological, chemical, and physical risks coexist, requiring comprehensive biosafety management. The handling of animal cadavers and biological specimens exposes students, instructors, and technical staff to potential infectious agents, toxic chemicals, and mechanical hazards associated with dissection procedures.

Cadavers used in anatomy teaching may contain viable microorganisms, including bacteria like *Enterococcus* spp., *E. coli*, and *S. aureus*, fungi like *Aspergillus* spp. and *Penicillium* spp., viruses, and parasites as previously described. As an example, although embalming significantly reduces the microbial load, complete elimination of pathogens is not guaranteed. Research has demonstrated the persistence of bacteria and fungi in paraffin-embedded tissues, and spore-forming organisms can survive preservation processes (Demiryurek et al. 2002; Tabaac et al. 2013; Brenner 2014). These observations come primarily from human cadaver literature and should be interpreted as indirect evidence when applied to veterinary anatomy. Furthermore, in human cadavers, infectious agents such as *Mycobacterium tuberculosis* and hepatitis viruses have historically been associated with the handling of human cadavers, particularly when donor screening and preservation procedures are inadequate (Shoja et al. 2013). Similar risks should be considered in animal cadavers, although direct comparative data specific to saline-preserved veterinary specimens are still scarce.

Chemical hazards represent another major danger in anatomy laboratories. Formaldehyde-based embalming solutions are widely used and are associated with acute irritant effects on the eyes and respiratory tract, as well as long-term carcinogenic risks. The International Agency for Research on Cancer (IARC) classifies formaldehyde as a Group 1 human carcinogen, and exposure in dissection rooms has been shown to exceed recommended occupational limits in poorly ventilated environments (Lyon 1994; Ohmichi et al. 2006). Consequently, ventilation systems, exposure monitoring, and alternative preservation techniques are increasingly emphasized in modern anatomy education.

Physical hazards are also prevalent in these environments. Sharp dissecting instruments, needles, and bone fragments pose a risk of cuts and puncture wounds, which can facilitate the transmission of pathogens. Ergonomic risks associated with prolonged standing, repetitive manual tasks, and manual handling of cadavers can contribute to musculoskeletal disorders among students and staff (Owolabi et al. 2022). Therefore, anatomy laboratories require integrated biosafety strategies that simultaneously address biological, chemical, and physical risks.

In general, anatomy laboratories should be recognized as multidisciplinary risk environments that require structured biosafety protocols, including personal protective equipment, ventilation control, waste management procedures, and biosafety training. Establishing such structures is essential to ensure safe educational practices and to comply with international occupational health and biosafety standards.

### Cadavers as potential reservoirs of microorganisms

Preserved cadavers should be kept under control as potential reservoirs of microorganisms, even when subjected to chemical fixation or alternative preservation techniques. Microbial populations can originate from the animal’s endogenous microbiota, the surrounding environment, or humans during transport, storage, and dissection procedures (Pereira et al. 2019; Ashfaq Ul Hassan 2022).

In veterinary contexts, the microbiological risk is further amplified by the possibility of zoonotic pathogens and antimicrobial-resistant strains. The persistence of these microorganisms in cadaveric tissues, embalming fluids, and laboratory surfaces has been reported, highlighting the relevance of cadavers as vectors of indirect exposure (CORREA 2003; Molina et al. 2019; Ashfaq Ul Hassan 2022). It is important to note that several of these cited studies were conducted on human cadavers (indirect evidence), while direct studies on saline-preserved veterinary cadavers are fewer.

Microbial indicators are commonly employed for biosafety assessment in anatomical and environmental studies. *Enterococcus* spp. are widely recognized as indicators of fecal contamination due to their high abundance in human and animal feces and notable persistence under adverse environmental conditions, including salinity, pH fluctuations, and desiccation (Byappanahalli et al. 2012; Boehm and Sassoubre 2014). *E. coli* serves as a standard marker of recent fecal contamination and can harbor antimicrobial resistance genes, posing additional public health risks through potential dissemination in the environment (Anjum et al. 2021; Nowicki et al. 2021). *S. aureus*, including methicillin-resistant strains, is associated with skin contact and fomite transmission, representing a significant risk during cadaver handling and anatomical dissection due to survival on surfaces and potential transfer to handlers (Kabadi et al. 2013; Jaradat et al. 2020).

In laboratories using saturated saline protection, halotolerant microorganisms can survive and persist despite hyperosmotic conditions. *Enterococci* and other salt-tolerant bacteria remained viable in high-salinity environments, emphasizing that saline preservation cannot be considered a sterilizing method (Torres et al. 2018). This highlights the need for continuous vigilance in biosecurity, particularly when adopting formaldehyde-free preservation techniques that prioritize technological realism over antimicrobial efficacy.

In general, the recognition of cadavers as microbiological reservoirs reinforces the need for biosecurity protocols, including routine microbiological monitoring, surface infection control, personal protective equipment, and environmental hygiene measures. These practices are essential to mitigate the risks of occupational exposure in both traditional and alternative anatomical preservation settings.

### Environmental contamination and fomite transmission

Laboratory surfaces and materials frequently act as fomites, indirectly transmitting microorganisms within anatomy facilities. Dissection tables, surgical instruments, gloves, protective clothing, and storage containers are repeatedly exposed to biological material and embalming fluids during practical classes, facilitating microbial dissemination via contact and aerosols. Several studies (Tabaac et al. 2013) have demonstrated that anatomy laboratory surfaces can become chronically contaminated and serve as reservoirs for opportunistic and potentially pathogenic microorganisms for opportunistic and potentially pathogenic microorganisms. The list include species like *S. aureus* (including methicillin-resistant strains, MRSA, in some reported cases), coagulase-negative staphylococci (*Staphylococcus epidermidis*, *S. hominis*, *S. warneri*), *Streptococcus pyogenes*, *Enterococcus faecalis*, Gram-negative bacilli such as *E. coli*, *Klebsiella pneumoniae* and other Enterobacterales, *Bacillus subtilis*, and fungi including *Aspergillus* spp., *Penicillium* spp., and other molds (Tabaac et al. 2013; Kosif and Avcioglu 2018; Gundreddy and Gaurkar 2022; Seçgin et al. 2024).

Cross-contamination is particularly relevant in teaching environments, where multiple classes of students share the same facilities for extended periods. Inadequate cleaning and disinfection protocols can lead to the accumulation of microbial biofilms on surfaces and equipment, increasing the risk of indirect exposure and occupational infection. Kosif and Avcioglu (Kosif and Avcioglu 2018) reported bacterial contamination on educational anatomical models and laboratory equipment, while Seçgin et al. (Seçgin et al. 2024) highlighted that inadequate hygiene practices contribute to environmental microbial dissemination in teaching laboratories.

Reusable instruments and materials represent a critical biosafety concern. Surgical instruments, scalpels, forceps, and probes can act as vectors for the transfer of microorganisms between cadavers and users if not properly sterilized. Similarly, gloves and personal protective equipment can be contaminated during handling and serve as vehicles for autoinoculation or environmental dissemination. Lab coats and aprons, often reused without proper washing, can accumulate microorganisms and contribute to persistent contamination (Kramer et al. 2006).

Storage containers and preservation solutions constitute another potential source of microbial persistence, particularly in laboratories that use alternative preservation methods such as saturated saline solution. Halotolerant bacteria and fungi can survive and proliferate in storage environments, highlighting the need for periodic solution changes and container decontamination. Environmental monitoring and standardized hygiene protocols are therefore essential to reduce the cumulative microbiological load in anatomy laboratories. Table 2 summarizes common fomites in anatomy laboratories, associated microbial contaminants, relative risk levels, and recommended hygiene practices based on empirical studies and established biosafety guidelines (Kramer et al. 2006; Richmond and McKinney 2009; Kosif and Avcioglu 2018; Torres et al. 2018; Seçgin et al. 2024).

**Table 2:** Common fomites in anatomy laboratories, associated contaminants, and recommended hygiene measures

Fomite	Typical Contaminants	Risk Level	Recommended Hygiene Measures
Dissection tables	*E. coli*,* Enterococcus* spp., environmental microorganisms	High	Daily surface disinfection with 70% ethanol, chlorine-based disinfectants, or quaternary ammonium compounds
Surgical instruments	*S. aureus*, Gram-negative bacilli, spore-forming bacteria	High	Autoclaving or validated chemical sterilization after each session
Gloves	*S. aureus*, environmental microorganisms	Moderate	Single-use gloves; discard after cadaver handling; hand hygiene after removal
Aprons/lab coats	*Enterococcus* spp., environmental fungi, skin associated-microorganisms	Moderate	Daily laundering or disposable garments; restricted laboratory use
Storage containers	Halotolerant bacteria, environmental fungi	Moderate	Weekly cleaning, periodic replacement of preservation solutions, container disinfection

Data compiled from CDC/NIH Biosafety in Microbiological and Biomedical Laboratories (BMBL) and environmental contamination studies ((Kramer et al. 2006; Richmond and McKinney 2009; Kosif and Avcioglu 2018; Torres et al. 2018; Seçgin et al. 2024)

### Microbiological monitoring protocols

Considering that most cadaver preservation methods do not sterilize cadavers, structured microbiological monitoring is considered a proposed best practice based on available evidence and expert interpretation, rather than a universally standardized protocol. It is intended to support biosafety in veterinary anatomy laboratories where formaldehyde-free preservation methods are used. Systematic surveillance enables early detection of contamination, guides cleaning and disinfection protocols, and ensures compliance with occupational safety standards.

The following monitoring components represent recommended precautionary measures derived from published studies and expert synthesis, due to the absence of fully standardized or evidence-based consensus protocols in veterinary anatomy settings. The main components of microbiological monitoring should include:

- **Cadaver surface sampling**: collecting samples from the cadaver surface (skin, oral cavity, viscera) at defined intervals provides information on the presence and diversity of microorganisms, including halotolerant bacteria, opportunistic fungi, and zoonotic agents. Culture-based methods and molecular techniques (PCR, qPCR, or sequencing) can be used for species identification and detection of antimicrobial resistance genes (Mudgal et al. 2022; Sebola et al. 2022);
- **Analysis of preservation solution**: saline solutions and other formaldehyde-free solutions should be sampled regularly to detect microbial growth, particularly halotolerant species such as *Enterococcus* spp. or *Staphylococcus* spp. Periodic testing guides decisions regarding solution replacement or the addition of low-risk antimicrobial agents (Kabadi et al. 2013);
- **Surveillance of laboratory surfaces and instruments**: dissection tables, instruments, storage containers, gloves, and lab coats often act as fomites, facilitating indirect microbial transmission. Routine collection of environmental samples and culture helps identify critical points of contamination, guiding targeted disinfection (Kramer et al. 2006; Seçgin et al. 2024);
- **Detection of zoonotic and antimicrobial-resistant pathogens**: veterinary cadavers can harbor pathogens, such as *Salmonella*,* Leptospira*,* Brucella*, or multidrug-resistant enteric bacteria. Targeted screening using selective media or molecular diagnostics can enhance risk assessment and support occupational health interventions (Sebola et al. 2022; Moreira Da Silva et al. 2025);
- **Data management and response protocols**: recording microbial load over time allows laboratories to track trends, assess the effectiveness of hygiene practices, and adjust monitoring frequency. Immediate interventions should be applied when indicator organisms or high-risk pathogens are detected, including enhanced disinfection, replacement of preservation solutions, or temporary suspension of the use of cadavers for teaching purposes.

The frequency of monitoring should be adapted to the intensity of laboratory use. For teaching environments with a high volume of samples, monthly sampling of cadavers and solutions is recommended, while facilities with lower usage may be sufficient with quarterly assessments (Varner et al. 2021). Integrated training for students and staff on proper sampling, use of personal protection equipment, and hygiene is essential to ensure accurate monitoring and minimize exposure risks. Combined monitoring strategies, encompassing cadaveric tissues, preservation solutions, and environmental surfaces, provide a comprehensive assessment of microbiological risks, particularly in formaldehyde-free environments where sterilization is not achieved (Sebola et al. 2022; Keiler et al. 2024; Seçgin et al. 2024). By implementing such protocols, veterinary anatomy laboratories can balance the educational benefits of preserved cadavers with occupational health safety, ensuring that educational objectives are met without compromising biosafety standards.

### Practical recommendations for handling saturated saline-preserved cadavers

The preservation method used in anatomy laboratories directly influences both occupational and microbiological biosafety conditions. Although formaldehyde-based fixation provides strong antimicrobial activity, alternative preservation methods such as saturated saline, alcohol-based, glycerin-based, or Thiel techniques generally present lower chemical toxicity while requiring greater attention to microbiological control measures. The following recommendations may help reduce biological and occupational risks associated with cadaver handling, particularly when non-sterilizing preservation methods are used, and are presented as proposed best practices intended to mitigate potential biological and occupational risks, particularly in the context of non-sterilizing preservation methods such as saturated saline solutions. These measures are based on available literature and biosafety principles but are not universally standardized guidelines:

i) Personal Protective Equipment (PPE): Appropriate PPE should be routinely used by all laboratory participants, including nitrile gloves (double gloving recommended during dissection), fluid-resistant laboratory coats or aprons, eye protection, and closed footwear. Face shields or masks are advisable during procedures with potential aerosol generation (Richmond and McKinney 2009; Epp and Waldner 2012);
ii) Cadaver Storage and Maintenance: Cadavers should be maintained according to the specific requirements of each preservation method. In saturated saline preservation, specimens should remain fully submerged in solutions containing ≥ 26% NaCl, with periodic replacement of the solution when turbidity or odor develops (Pereira et al. 2019). Formaldehyde-fixed specimens require adequate ventilation and monitoring of airborne formaldehyde concentrations (Zdilla 2021);
iii) Dissection Procedures: Practical sessions should ideally be limited to 2–3 h in order to minimize prolonged exposure. The use of downdraft tables or local exhaust ventilation is recommended, particularly during procedures capable of generating aerosols or chemical exposure;
iv) Environmental Hygiene and Decontamination: Dissection tables, instruments, and laboratory surfaces should be disinfected after each session using 70% ethanol, chlorine-based disinfectants, or equivalent validated agents. Routine hand hygiene practices should be strictly reinforced (Kosif and Avcioglu 2018; Seçgin et al. 2024);
v) Microbiological Monitoring: Periodic microbiological surveillance of cadavers, preservation solutions, and laboratory surfaces is particularly important when non-sterilizing preservation methods such as saturated saline, alcohol-based, or glycerin-based techniques are employed (Pereira et al. 2019; Seçgin et al. 2024);
vi) Waste Management: Cadaveric tissues, fluids, and contaminated materials should be treated as potentially biohazardous and discarded according to institutional biosafety regulations and local legislation (Richmond and McKinney 2009).

## Occupational exposure risks and mitigation strategies

Occupational exposure in veterinary anatomy laboratories involves chemical, biological, and physical risks associated with the handling, preservation, and dissection of cadavers. Although several preservation techniques aim to reduce the toxicity associated with traditional formaldehyde-based fixation, alternative preservation methods are often non-sterilizing and may permit the persistence of microorganisms, potentially increasing the risks of biological exposure during laboratory activities (Lombardero et al. 2017; Pereira et al. 2019).

Chemical hazards remain a relevant concern in anatomy laboratories. Residual chemical exposure may occur in hybrid preservation protocols or when trace amounts of formaldehyde remain in combined fixation approaches. Even at low concentrations, formaldehyde can cause mucosal irritation and respiratory symptoms and is classified as a Group 1 carcinogen by the International Agency for Research on Cancer (Ohmichi et al. 2006). Consequently, facilities adopting alternative preservation methods, including saline-based systems, should maintain adequate ventilation systems and implement environmental monitoring to control potential exposure to residual vapors. Biological risks mainly stem from microbial presence in preserved cadavers.

Microbiological analyses of canine cadavers preserved with ethyl alcohol followed by saturated saline solution have demonstrated the survival of microorganisms such as spore-forming and halotolerant *Bacillus* spp. over extended periods, generally at low microbial loads (Pereira et al. 2019). Although severe laboratory-acquired infections from preserved cadavers are relatively uncommon, several incidents and near-misses illustrate the importance of proper protocols. Pereira et al. (2019) observed a progressive increase in halotolerant bacterial counts (particularly *Bacillus* spp. and *Enterococcus* spp.) in canine cadavers when saline solutions were not regularly replaced, leading to visible cloudiness and mild skin irritation among students after prolonged contact. In human anatomy laboratories, inadequate PPE and poor hand hygiene have been linked to colonization or infection by *S. aureus* and *Enterococcus* spp. among students and staff (Kosif and Avcioglu 2018; Ashfaq Ul Hassan 2022). Similar risks exist in veterinary settings, particularly when cadavers originate from animals with subclinical infections. Cases of allergic reactions and respiratory symptoms associated with fungal overgrowth (*Aspergillus* and *Penicillium* spp.) have also been reported in environments with high humidity and insufficiently maintained saline-preserved specimens (Molina et al. 2019). In addition to microbial exposure, occupational allergies may develop following repeated contact with animal proteins, tissues, or preservation chemicals. These reactions may manifest as respiratory or dermatological symptoms and may require adjustments to laboratory protocols or personal protective measures (Epp and Waldner 2012).

Physical hazards are also common in anatomy laboratories. Injuries associated with sharp dissection instruments, accidental punctures, and ergonomic strain resulting from prolonged cadaver handling represent frequent occupational risks. Such injuries may be complicated by secondary infections if adequate wound management is not implemented promptly (Owolabi et al. 2022). Risk mitigation strategies typically follow a hierarchical approach that prioritizes engineering controls, administrative measures, and personal protective equipment (PPE). Engineering controls include adequate ventilation systems, fume extraction tables, and, when appropriate, Class II biological safety cabinets for procedures that may generate aerosols. Administrative measures involve mandatory training in safe cadaver handling, method-specific risk assessments, standardized laboratory protocols, and immediate wound-care procedures. The consistent use of appropriate PPE, including nitrile gloves, protective gowns, face shields, and respirators such as N95 masks in aerosol-generating situations, further reduces occupational exposure risks.

In addition, occupational health surveillance programs, including allergy monitoring and vaccination against relevant zoonotic diseases when applicable, can support early identification and management of potential health risks among laboratory personnel and students (Epp and Waldner 2012; Owolabi et al. 2022). The integrated implementation of these strategies supports the safe and sustainable adoption of alternative preservation methods while ensuring compliance with occupational health standards and protecting personnel in veterinary teaching environments.

## Regulatory frameworks and best practices

Biosafety management in veterinary anatomy teaching laboratories using preserved animal cadavers is guided by a combination of international standards and national regulatory frameworks aimed at minimizing biological and occupational risks. The World Organization for Animal Health (WOAH) provides overarching principles for biohazard management in veterinary laboratories, emphasizing systematic risk assessment, implementation of biosafety levels, and containment strategies tailored to the biological agents potentially present in animal specimens (Health 2018). These principles are particularly relevant to gross anatomy laboratories, where the risk profile differs from diagnostic or research animal facilities due to the repeated, long-term handling of the same preserved specimens by large groups of students. These recommendations highlight the importance of formal biological risk management systems designed to prevent accidental exposure or environmental release of pathogens.

Complementary guidance is provided by the Centers for Disease Control and Prevention (CDC) and the National Institutes of Health (NIH) through the Biosafety in Microbiological and Biomedical Laboratories (BMBL) manual, which defines Animal Biosafety Levels (ABSL-1 to ABSL-4) applicable to facilities handling animal tissues or potentially infectious materials (Richmond and McKinney 2009). In the specific context of veterinary anatomy laboratories with cadavers preserved in saturated saline solution, a non-sterilizing method that allows survival of halotolerant microorganisms, the BMBL guidelines are applied by classifying most activities under ABSL-1 or ABSL-2. Within veterinary anatomy teaching laboratories, preserved cadavers are generally managed under conditions comparable to ABSL-1 or ABSL-2, depending on the origin of the specimens and the likelihood of exposure to zoonotic microorganisms. ABSL-1 is typically adequate for cadavers from healthy animals preserved in saturated saline when strict hygiene and monitoring protocols are followed. ABSL-2 practices become necessary when there is a reasonable risk of zoonotic agents (for example, *Salmonella* spp., *L. monocytogenes*, or antimicrobial-resistant bacteria) or when handling fresh tissues or opening body cavities that may generate bioaerosols. Recommended practices include controlled laboratory access, appropriate personal protective equipment (PPE), and the implementation of engineering controls to reduce potential exposure to biological hazards. In saline-preserved cadavers, particular attention is given to local exhaust ventilation (for example, downdraft tables) not only for chemical vapors but also to control potential bioaerosols generated during prolonged dissection sessions. The BMBL also emphasizes the importance of secondary containment barriers, including architectural and infrastructural features such as self-closing doors, hands-free sinks, and appropriate laboratory layout designed to reduce cross-contamination and improve hygiene practices within teaching environments (Richmond and McKinney 2009).

Furthermore, unlike diagnostic or research facilities that usually handle single-use specimens under short-term workflows, anatomy teaching laboratories involve repeated student contact with the same cadavers over weeks or months. This reality requires reinforced biosafety and hygiene practices during repeated dissection sessions.

## Knowledge gaps and future research directions

Despite advances in cadaver preservation, substantial knowledge gaps remain regarding the long-term biosafety of preservation methods in veterinary anatomy education. Existing literature emphasizes tissue quality and short-term microbial persistence, with limited longitudinal data on pathogen viability and transmission dynamics across different preservatives (de Oliveira 2014; Lombardero et al. 2017). Some studies report minimal contamination over extended periods, whereas others identify the presence of halotolerant or environmental microorganisms, suggesting that microbial outcomes may vary depending on cadaver origin, preservation formulation, storage conditions, and handling practices (Pereira et al. 2019).

A further limitation is the lack of standardized biosafety protocols in veterinary anatomy laboratories. Differences in handling procedures, PPE use, ventilation, and monitoring practices make comparisons between institutions difficult and may affect contamination levels. In addition, relatively little research has addressed environmental contamination within anatomy laboratories, including microbial persistence on dissection tables, instruments, and frequently touched surfaces, as well as the potential generation of airborne particles during dissection activities.

A further gap concerns the quantification of occupational exposure risks associated with cadaver handling. Prospective cohort studies assessing the incidence of infections, allergies, or other health outcomes among laboratory personnel and students are scarce, limiting the development of evidence-based occupational safety guidelines. This issue may be particularly relevant in veterinary settings, where cadavers can originate from clinical or field environments and may harbor zoonotic microorganisms or opportunistic pathogens. The potential presence and persistence of antimicrobial-resistant microorganisms in preserved cadavers or laboratory environments also remains poorly characterized and warrants systematic investigation. Furthermore, the efficacy of adjunct antimicrobial strategies incorporated into preservation solutions remains underexplored. Hybrid preservation approaches combining traditional fixatives with targeted antimicrobial agents may offer opportunities to improve microbial control while minimizing toxicity and preserving tissue quality (de Oliveira 2014). Future research should prioritize multicenter studies implementing standardized monitoring protocols, enabling robust comparisons of microbiological outcomes across preservation methods. The application of advanced analytical techniques, such as metagenomic sequencing, could further improve the comprehensive characterization of microbial communities associated with preserved cadavers and laboratory environments. Comparative investigations integrating microbiological, chemical, and biomechanical parameters would help identify optimal preservation strategies that balance educational value, biosafety, and occupational health. Finally, the development of sustainable and environmentally friendly preservation techniques, aligned with evolving safety regulations and reduced chemical exposure, represents an important direction for future innovation in veterinary anatomy education (Hayashi et al. 2016). Addressing these knowledge gaps will contribute to the safe implementation of emerging preservation methods, while supporting veterinary training and protecting the health of students, instructors, and laboratory personnel. A further limitation is the scarcity of quantitative microbiological data. Most published studies report qualitative or semi-qualitative findings (presence/absence of specific genera), with few providing longitudinal colony-forming unit counts or direct comparisons between preservation methods. This lack of robust quantitative information makes it difficult to precisely determine the relative microbiological risk of saturated saline versus other techniques over extended periods.

Although this review provides a structured synthesis of the available evidence on preservation methods in veterinary anatomy, several limitations should be acknowledged. First, the available literature on microbiological outcomes, particularly for alternative preservation techniques such as saturated saline solutions, remains limited and heterogeneous. Second, a substantial proportion of the microbiological evidence is derived from human anatomy or experimental studies, requiring cautious extrapolation to veterinary settings. Third, variability in study design, microbiological assessment methods, and reporting standards limits direct comparability across studies. Finally, the absence of standardized protocols for microbiological monitoring in anatomy laboratories restricts the ability to draw definitive conclusions. These limitations highlight the need for more systematic and longitudinal studies in this field.

## Conclusion

Cadaver-based dissection remains a cornerstone of veterinary anatomy education, providing tactile and three-dimensional learning experiences that digital tools cannot fully replicate. However, the preservation methods used to maintain cadaveric material have important implications for both educational quality and laboratory biosafety. Traditional formaldehyde-based fixation offers reliable long-term preservation and broad antimicrobial efficacy, but its well-documented occupational hazards including mucosal irritation and carcinogenic potential, have prompted increasing interest in alternative preservation strategies.

Formaldehyde-free fixation methods, such as alcohol-, glycerin-, Thiel-, or saturated saline - based techniques, potentially improve tissue flexibility, color retention, and overall realism, although the evidence supporting saturated saline remains limited and should be interpreted cautiously. While pedagogical and chemical safety advantages are supported by veterinary studies, microbiological evidence remains less consolidated and often relies on extrapolation from human anatomy or environmental microbiology. The main conceptual contribution of this review is the integration of three distinct evidence domains, educational realism, occupational chemical safety, and microbiological biosafety, which are rarely analyzed together. By applying this framework specifically to saturated saline preservation in veterinary anatomy, this work provides a practical model for evaluating trade-offs when adopting formaldehyde-free preservation methods. This multi-dimensional approach helps move the field beyond isolated technical descriptions toward more informed decision-making in veterinary education. Adopting alternative preservation strategies requires integration of biosafety measures to ensure safe veterinary anatomy education. When properly implemented, these measures can effectively improve laboratory safety while maintaining safe learning environments.

Adopting alternative preservation strategies, when combined with robust biosafety measures, helps balance pedagogical quality with occupational safety. Several recommendations regarding microbiological monitoring and laboratory practices should be interpreted as precautionary, evidence-informed proposals rather than established consensus guidelines Future research should prioritize longitudinal studies on microbial survival in preserved cadavers, comparative evaluations of biosafety outcomes across preservation techniques, and the development of hybrid or environmentally sustainable preservation methods. Integrating innovative preservation strategies with robust biosafety practices will enable veterinary institutions to maintain high-quality anatomical training while protecting the health of students, instructors, and laboratory personnel.

## Data Availability

No new datasets were generated or analyzed during the current study. All data discussed are included within the article and its cited references.

## References

[CR1] Abdullah E, Lone M, Cray JJ, Dvoracek P, Balta JY (2021) Medical students’ opinions of anatomy teaching resources and their role in achieving learning outcomes. Med Sci Educ 31:1903–1910. 10.1007/s40670-021-01436-2 34950529 PMC8651893

[CR2] Anjum MF, Schmitt H, Borjesson S, Berendonk TU (2021) The potential of using *e. coli* as an indicator for the surveillance of antimicrobial resistance (AMR) in the environment. Curr Opin Microbiol 64:152–158. 10.1016/j.mib.2021.09.011 34739920

[CR3] Ashfaq Ul Hassan SH, Azhar S (2022) The potential risk of microbial transmission from cadavers to its handlers including teaching staff, medical students, paramedical staff working in dissection hall. Int J Med Res Health Sci 11:1–8. 10.14704/nq.2022.20.12.NQ77064

[CR4] Balta JY, Twomey M, Moloney F, Duggan O, Murphy KP, O’Connor OJ, Cronin M, Cryan JF, Maher MM, O’Mahony SM (2019) A comparison of embalming fluids on the structures and properties of tissue in human cadavers. Anat Histol Embryol 48:64–73. 10.1111/ahe.12412 30450564

[CR5] Boehm AB, Sassoubre LM (2014) Enterococci as indicators of environmental fecal contamination. In: Gilmore MS, Clewell DB, Ike Y, Shankar N (eds) Enterococci: From commensals to leading causes of drug resistant infection. Boston 24649503

[CR6] Brenner E (2014) Human body preservation–old and new techniques. J Anat 224:316–344. 10.1111/joa.12160 24438435 PMC3931544

[CR7] Byappanahalli MN, Nevers MB, Korajkic A, Staley ZR, Harwood VJ (2012) Enterococci in the environment. Microbiol Mol Biol Rev 76:685–706. 10.1128/MMBR.00023-12 23204362 PMC3510518

[CR8] Choudhary OP, Sarkar R (2025) Animal anatomical teaching models for enhanced veterinary anatomy education and learning. Pak Vet J. 10.29261/pakvetj/2025.250

[CR9] Clifton W, Damon A, Nottmeier E, Pichelmann M (2020) The importance of teaching clinical anatomy in surgical skills education: spare the patient, use a sim! Clin Anat 33:124–127. 10.1002/ca.23485 31581311

[CR10] CORREA WR (2003) Isolamento e identificação de fungos filamentosos encontrados em peças anatômicas conservadas em solução de formol a 10%. Instituto de Pesquisa e Desenvolvimento

[CR11] Da Silva RMG, Matera JM, Ribeiro AACM (2004) Preservation of cadavers for surgical technique training. Vet Surg 33:606–608. 10.1111/j.1532-950x.2004.04083.x 15659015

[CR12] de Oliveira FS (2014) Assessing the effectiveness of 30% sodium chloride aqueous solution for the preservation of fixed anatomical specimens: a 5-year follow-up study. J Anat 225:118–121. 10.1111/joa.12185 24762210 PMC4089352

[CR13] Demiryurek D, Bayramoglu A, Ustacelebi S (2002) Infective agents in fixed human cadavers: a brief review and suggested guidelines. Anat Rec 269:194–197. 10.1002/ar.10143 12209557

[CR14] Eisma R, Lamb C, Soames RW (2013) From formalin to Thiel embalming: what changes? One anatomy department’s experiences. Clin Anat 26:564–571. 10.1002/ca.22222 23408386

[CR15] Epp T, Waldner C (2012) Occupational health hazards in veterinary medicine: zoonoses and other biological hazards. Can Vet J 53:144–150 22851775 PMC3258827

[CR16] Fox CH, Johnson FB, Whiting J, Roller PP (1985) Formaldehyde fixation. J Histochem Cytochem 33:845–853. 10.1177/33.8.3894502 3894502

[CR17] Ghosh SK (2015) Human cadaveric dissection: a historical account from ancient Greece to the modern era. Anat Cell Biol 48:153–169. 10.5115/acb.2015.48.3.153 26417475 PMC4582158

[CR18] Gundreddy P, Gaurkar SS (2022) Retracted: presence of contagious bacterial flora in formalin-fixed cadavers: a potential health hazard to medical professionals. Cureus. 10.7759/cureus.30684 36439597 PMC9691388

[CR19] Handady G, Dsouza A, Nayak V, Abraham J (2024) Formaldehyde levels and the indoor air quality of an anatomy dissection hall with different ventilation setups. Environ Health Insights 18:11786302241301590. 10.1177/11786302241301590 39610457 PMC11603455

[CR20] Hayashi S, Naito M, Kawata S, Qu N, Hatayama N, Hirai S, Itoh M (2016) History and future of human cadaver preservation for surgical training: from formalin to saturated salt solution method. Anat Sci Int 91:1–7. 10.1007/s12565-015-0299-5 26670696

[CR21] Health WOA (2018) Biosafety and biosecurity: Standard for managing biological risk in the veterinary laboratory and animal facilities. WOAH Terrestrial Manual 2018

[CR22] Jaradat ZW, Ababneh QO, Sha’aban ST, Alkofahi AA, Assaleh D, Al Shara A (2020) Methicillin resistant *Staphylococcus aureus* and public fomites: a review. Pathog Glob Health 114:426–450. 10.1080/20477724.2020.1824112 33115375 PMC7759291

[CR23] Kabadi CJ, Smith CR III, Gomez F (2013) Potential pathogen transmission on medical student anatomy laboratory clothing. Med Stud Res J 2:30–35. 10.15404/msrj.002.002.spring/04

[CR24] Kaliappan A, Motwani R, Gupta T, Chandrupatla M (2023) Innovative cadaver preservation techniques: a systematic review. Maedica (Bucur) 18:127–135. 10.26574/maedica.2023.18.1.127 37266469 PMC10231151

[CR25] Keiler J, Bast A, Reimer J, Kipp M, Warnke P (2024) Quantitative and qualitative assessment of airborne microorganisms during gross anatomical class and the bacterial and fungal load on formalin-embalmed corpses. Sci Rep 14:19061. 10.1038/s41598-024-69659-y 39154062 PMC11330451

[CR26] Kosif R, Avcioglu F (2018) An examination of bacterial contamination of models used in anatomy laboratories. Interdiscip Perspect Infect Dis 2018:9201312. 10.1155/2018/9201312 30662459 PMC6313981

[CR27] Kramer A, Schwebke I, Kampf G (2006) How long do nosocomial pathogens persist on inanimate surfaces? A systematic review. BMC Infect Dis 6:130. 10.1186/1471-2334-6-130 16914034 PMC1564025

[CR28] Kwizera R, Naluzze J (2024) A systematic review of fungi isolated from formalin-preserved human and animal cadavers: a potential health concern to exposed students and technicians. IJID One Health 4:100037. 10.1016/j.ijidoh.2024.100037

[CR29] Latorre RM, Garcia-Sanz MP, Moreno M, Hernandez F, Gil F, Lopez O, Ayala MD, Ramirez G, Vazquez JM, Arencibia A, Henry RW (2007) How useful is plastination in learning anatomy? J Vet Med Educ 34:172–176. 10.3138/jvme.34.2.172 17446645

[CR30] Latorre R, Bainbridge D, Tavernor A, Lopez Albors O (2016) Plastination in anatomy learning: an experience at Cambridge university. J Vet Med Educ 43:226–234. 10.3138/jvme.0715-113R1 27075277

[CR31] Lingnau E (1957) Das verhalten der werkstoffe gegenüber formaldehyd. Mater Corros 8:480–487. 10.1002/maco.19570080806

[CR32] Lombardero M, Yllera MM, Costa ESA, Oliveira MJ, Ferreira PG (2017) Saturated salt solution: a further step to a formaldehyde-free embalming method for veterinary gross anatomy. J Anat 231:309–317. 10.1111/joa.12634 28542788 PMC5522894

[CR33] Lyon F (1994) Iarc monographs on the evaluation of carcinogenic risks to humans. Some industrial chemicals 60:389–433 PMC7681540 7869568

[CR34] Manero A, Blanch AR (1999) Identification of *Enterococcus* spp. with a biochemical key. Appl Environ Microbiol 65:4425–4430. 10.1128/AEM.65.10.4425-4430.1999 10508070 PMC91588

[CR35] Molina C, Berrocal L, Jofré MR, Rosas C, Rojas X (2019) Identification of bacterial and fungal species in human cadavers used in anatomy teaching. 37(2):473–476. 10.4067/S0717-95022019000200473

[CR36] Moreira da Silva J, Menezes J, Fernandes L, Marques C, Costa SS, Timofte D, Amaral AJ, Pomba C (2025) Evaluation of multidrug-resistant bacteria and their molecular mechanisms found in small animal veterinary practices in Portugal. Front Cell Infect Microbiol 15:1582411. 10.3389/fcimb.2025.1582411 40391047 PMC12086164

[CR37] Mudgal S, Dabral S, Juyal D, Thakur A (2022) Microbial contamination of the anatomical models used in the museum and dissection hall of the department of anatomy. J Pharm Sci Res 14:748–752

[CR38] Nam SM, Moon JS, Yoon HY, Chang BJ, Nahm SS (2020) Comparative evaluation of canine cadaver embalming methods for veterinary anatomy education. Anat Sci Int 95:498–507. 10.1007/s12565-020-00547-x 32356264

[CR39] Nowicki S, deLaurent ZR, de Villiers EP, Githinji G, Charles KJ (2021) The utility of *Escherichia coli* as a contamination indicator for rural drinking water: evidence from whole genome sequencing. PLoS One 16:e0245910. 10.1371/journal.pone.0245910 33481909 PMC7822521

[CR40] Ohmichi K, Komiyama M, Matsuno Y, Takanashi Y, Miyamoto H, Kadota T, Maekawa M, Toyama Y, Tatsugi Y, Kohno T (2006) Formaldehyde exposure in a gross anatomy laboratory. Personal exposure level is higher than indoor concentration (5 pp). Environ Sci Pollut Res 13:120–124. 10.1065/espr2005.06.265 16612901

[CR41] Owolabi JO, Tijani AA, Ihunwo AO (2022) A need to protect the health and rights of anatomists working in dissection laboratories. Risk Manag Healthc Policy 15:889–893. 10.2147/RMHP.S362305 35547644 PMC9081885

[CR42] Pereira N, Cardozo MV, de Sá Rocha TAS, Zero RC, De Ávila FA, De Oliveira FS (2019) Microbiological analysis in the fixation and preservation of dog cadavers with ethyl alcohol and sodium chloride solution análise microbiológica na fixação e conservação de cadáveres de cães com álcool etílico e solução de cloreto de sódio. Semina Cienc Agrar Londrina 40:3099–3106. 10.5433/1679-0359.2019vv40n6Supl2p3099

[CR43] Persaud TV, Loukas M, Tubbs RS (2014) A history of human anatomy. Charles C. Thomas, Publisher, Limited

[CR44] Richmond JY, McKinney RW (2009) Biosafety in microbiological and biomedical laboratories. US Government Printing Office

[CR45] Sebola DC, Oguttu JW, Kock MM, Qekwana DN (2022) Hospital-acquired and zoonotic bacteria from a veterinary hospital and their associated antimicrobial-susceptibility profiles: a systematic review. Front Vet Sci 9:1087052. 10.3389/fvets.2022.1087052 36699325 PMC9868922

[CR46] Seçgin Y, Toy Ş, Solmaz H, Başar E (2024) Analysis of an anatomy laboratory for microbiological contamination. Northwest Med J 4:106–112. 10.54307/2024.NWMJ.114

[CR47] Senos R (2024) Plastinate library: a tool to support veterinary anatomy learning. Animals (Basel) 14:223. 10.3390/ani14020223 38254392 PMC10812824

[CR48] Shoja MM, Benninger B, Agutter P, Loukas M, Tubbs RS (2013) A historical perspective: infection from cadaveric dissection from the 18th to 20th centuries. Clin Anat 26:154–160. 10.1002/ca.22169 23037893

[CR49] Streich S, Ladurner R, Grashorn SM, Liese J, Hirt B, Neckel PH (2025) Exploring entry pathways of microorganisms into an anatomical dissection course. Sci Rep. 10.1038/s41598-025-30667-1 41331037 PMC12675709

[CR50] Tabaac B, Goldberg G, Alvarez L, Amin M, Shupe-Ricksecker K, Gomez F (2013) Bacteria detected on surfaces of formalin fixed anatomy cadavers. Ital J Anat Embryol 118:1–5 23898574

[CR51] Tamayo-Arango L, Garzón-Alzate A (2018) Preservation of animal cadavers with a formaldehyde-free solution for gross anatomy. J Morphol Sci 35:136–141. 10.1055/s-0038-1669434

[CR52] Terrado J, Gomez O, Chicharro D, Garcia-Manzanares M, Juarez M, Romo-Barrientos C, Mohedano-Moriano A, Criado-Alvarez JJ (2023) Anxiety, emotions, and thoughts of veterinary medicine students during their first visit to the dissection room. Anat Sci Educ 16:547–556. 10.1002/ase.2258 36695649

[CR53] Thiel W (1992) Die konservierung ganzer leichen in natürlichen farben. Ann Anat 174:185–195 1503236

[CR54] Torres C, Alonso CA, Ruiz-Ripa L, Leon-Sampedro R, Del Campo R, Coque TM (2018) Antimicrobial resistance in *Enterococcus* spp. of animal origin. Microbiol Spectr 6:185. 10.1128/microbiolspec.ARBA-0032-2018 PMC11633606 30051804

[CR55] Varner C, Dixon L, Simons MC (2021) The past, present, and future: a discussion of cadaver use in medical and veterinary education. Front Vet Sci 8:720740. 10.3389/fvets.2021.720740 34859081 PMC8631388

[CR56] Waschke J, Bergmann M, Brauer L, Brenner E, Buchhorn A, Deutsch A, Dokter M, Egu DT, Ergun S, Fassnacht U, Fietz D, Gundlach S, Heermann S, Hirt B, Kugelmann D, Muller-Gerbl M, Neiss W, Nimtschke U, Plendl J, Pretterklieber M, Redies C, Scaal M, Schmidt MHH, Schmiedl A, Schnittler HJ, Schomerus C, Sebesteny T, Spittau B, Steiniger B, Tschernig T, Unverzagt A, Viebahn C, Voigt E, Weigner J, Weyers I, Winkelmann A, Winkler M, Paulsen F (2019) Recommendations of the working group of the anatomische gesellschaft on reduction of formaldehyde exposure in anatomical curricula and institutes. Ann Anat 221:179–185. 10.1016/j.aanat.2018.10.007 30393181

[CR57] Yunus HA, Ekim O, Bakıcı C, Batur B, Çakır A (2022) From toxic cadavers to biosafe specimens: a brief history of plastination in veterinary anatomy. J Turk Vet Med Soc 93:158–165. 10.33188/vetheder.998978

[CR58] Zdilla MJ (2021) Local exhaust ventilation systems for the gross anatomy laboratory. Morphologie 105:237–246. 10.1016/j.morpho.2020.11.002 33279395 PMC8169711

